# Photosynthetic Efficiency and Proteome Response of Diploid and Polyploid *Arabidopsis thaliana* After Heat or Salt Stress

**DOI:** 10.3390/genes16111278

**Published:** 2025-10-28

**Authors:** Nenad Malenica, Roko Gvozdenica Šipić, Anamaria Đerek, Jelena Mlinarec, Mirta Tkalec, Dubravko Pavoković

**Affiliations:** 1 Division of Molecular Biology, Department of Biology, Faculty of Science, University of Zagreb, 10000 Zagreb, Croatia; malenica@biol.pmf.unizg.hr (N.M.); roko.gvozdenicasipic@unibas.ch (R.G.Š.); a.derek@unibas.ch (A.Đ.); jelena@oikon.hr (J.M.); mirta.tkalec@biol.pmf.unizg.hr (M.T.); 2 Biozentrum, University of Basel, 4056 Basel, Switzerland; 3 Institute of Applied Ecology, Oikon Ltd., 10020 Zagreb, Croatia

**Keywords:** Arabidopsis, tetraploid, triploid, heat stress, salt stress, proteome, maximum photosynthetic efficiency, proline

## Abstract

Global warming and soil salinization pose significant challenges to modern plant cultivation. Background/Objectives: Polyploidization of whole-genome duplication is an important evolutionary strategy, enhancing plant adaptation to environmental stress. This study investigates the impact of heat and salt stress on photosynthesis and proteomic changes in a polyploid series of *Arabidopsis thaliana* (diploid, triploid, and tetraploid). Methods: Two-month-old plants were exposed to heat stress (45 °C for 3 h) or salt stress (300 mM NaCl for 24 or 48 h). Stress effects were assessed via photosystem II maximum efficiency (*F_v_/F_m_*), the performance index (*PI_ABS_*), and proline content. Proteomic responses were analyzed using 2D SDS-PAGE and mass spectrometry. Results: Our findings revealed that polyploid plants maintained higher photosynthetic performance than diploids under both heat and salt stress. While proline accumulation under heat stress was comparable across all ploidy levels, polyploids accumulated more proline under salt stress, indicating enhanced salinity tolerance. Proteomic analysis showed differential protein expression among diploid and polyploid plants in response to stress. Several differentially expressed proteins had functions involved in photosynthesis and stress response pathways. These findings confirm prior evidence of tetraploid Arabidopsis resilience to salinity and extend this observation to heat stress. Moreover, triploids also demonstrated increased stress tolerance, suggesting adaptive advantages of this intermediate ploidy level as well. Conclusions: Differential expression patterns among ploidy levels may reflect varied energy-saving strategies and alterations in protein structure and function. This work highlights the importance of polyploidy in improving plant stress resilience, offering insights for breeding stress-tolerant crops in a changing climate.

## 1. Introduction

It is rather a common observation that polyploid flowering plants have a higher capacity to cope with a variety of biotic stressors. The advent and proliferation of polyploid plants seem to coincide with environmental upheavals leading to the mass extinction event at the Cretaceous–Paleogene boundary (65 million years ago), suggesting their high adaptive potential in the newly formed environmental conditions [1,2,3]. Moreover, stressful stimuli increase the chance of polyploid generation. Specifically, stressors like high temperature have a negative influence on synaptonemal complex formation and chromosome segregation, leading to the formation of unreduced gametes [4,5], a prerequisite for triploid or tetraploid offspring. Also, high temperatures can lead to increased frequencies of haploid offspring in systems with CENH3 centromeric histone incompatibility [6]. Heat impairs CENH3 levels at the centromere and therefore wakens the kinetochore function [7]. In addition, the formation of triploids by fusion of haploid and unreduced gametes is an important step towards the establishment of tetraploids. Namely, in Arabidopsis, triploids are fertile and generate predominantly aneuploids, as well as diploids, triploids, and tetraploids upon selfing [8]. Due to climate change, the two most prominent stressors are high temperature and salinity, the latter due to low soil quality or intensive soil irrigation, very often acting in combination [9]. Salt stress affects plants in two primary ways: ionic and osmotic. The osmotic effect leads to reduced water uptake by the plant, while the ionic effect causes intracellular toxicity and disruption of homeostasis [10]. There are also secondary effects at the cellular level, such as oxidative stress, damage to membrane lipids, proteins, and nucleic acids, and various disruptions in metabolic functions [11]. During salt stress on 300 mM NaCl, diploid soil-grown Arabidopsis plants accumulate proline in order to stabilize cellular homeostasis [12,13]. It enhances stress tolerance by acting as an osmolyte, a scavenger of ROS, a source of nitrogen and carbon for stress-affected cells, and a pH buffer [14].

Heat stress occurs when a plant grows at temperatures above the optimal range. Heat stress primarily affects components of the cellular machinery responsible for photosynthesis, specifically photosystem II, RUBISCO, and ATP synthase [15]. While photosynthesis inhibition due to moderate stress is reversible, temperatures above 40 °C cause irreversible damage, including permanent denaturation of proteins [16]. To achieve a strong stress response, a 45 °C heat shock for 3 h is sufficient [17].

There are many instances where tetraploid crop plants perform better under heat or salinity stress. For example, 4n apples perform better under high-salinity conditions. Their roots retain more water and express more aquaporins in their membranes [18]. Tetraploid rice tolerates high salinity by reducing Na⁺ uptake and, in general, possesses more stable membranes and organelles [19]. Also, in a similar setting, tetraploid rice shows increased jasmonate (JA) signaling and accumulates JA conjugates, making it more resilient under salt stress conditions [20]. Further, watermelon tetraploid rootstock was sufficient to mitigate salinity stress in a diploid scion in terms of a better Na^+^/K^+^ ratio and better photosynthetic parameters [21]. Also, several tetraploid citrus species, like orange [22] and mandarin [23,24], accumulated more antioxidative enzymes and proline, showing less leaf damage, as well as accumulating more chlorophyll and K^+^, respectively. On the other hand, there are examples where polyploids under stress perform worse than diploids. For example, diploid sugar beet tolerated salt stress better than its triploid and tetraploid cytotypes [25]. Also, several allopolyploid cottons species were less tolerant to salt stress than diploids [26]. In Arabidopsis, polyploids up to octoploids have been recently extensively phenotypically described. Tetraploid plants are bigger and more vigorous than diploids, unlike 6n and 8n plants, which have comparatively smaller rosettes. In addition, all polyploids show a thinner secondary cell wall with less lignin and cellulose content, weakening the structural stability of plant tissues and leading to vessels collapse [27]. At the same time, octoploid Arabidopsis cells were shown to be bigger, therefore having a larger storing potential leading to more resilience to nutrient deprivation when it arises [28]. However, these were not stress-related studies. Unlike even-number polyploids, triploids are underused as a stress-related research cytotype, although some triploid crop plants were demonstrated to be commercially useful (for example, Cavendish banana, Summer Sweet watermelon, or Jonagold apple [29]). In several crop species, triploids can outperform diploid or tetraploid counterparts under stress conditions, like the horticultural species *Angelonia angustifolia,* which has more flowers and more branches and flowers early under elevated heat [30], or like triploid poplar, which better tolerates salinity stress [31]. In Arabidopsis, the predominant polyploid cytotype used in stress mitigation research is 4n. For instance, in a study on salinity effects on tetraploids, it was observed that 4n plants of various accessions cope with stress better than 2n plants, due to intracellular K^+^ accumulation. Also, stressed 4n plants had a smaller reduction in fecundity in comparison to 2n [32]. However, other groups showed negative effects in tetraploid Arabidopsis under stress. For example, it was found that tetraploids were more susceptible to glucose-induced osmotic stress than diploids [33]. Also, homologous chromosome pairing was impaired at elevated temperatures, leading to the formation of more univalents at metaphase I [34]. As a consequence, heat stress eventually leads to less pollen production [5]. Finally, there are transcriptomic or proteomic studies focusing on global stress responses of polyploids in particular. A study showed that Arabidopsis transcription in tetraploids vs. diploids is ecotype-specific and that Columbia accession showed more than four hundred differentially expressed genes in the tetraploid, whereas Landsberg showed only nine transcripts that were differentially expressed [35]. Also, genes were only slightly up- (1.5×) or downregulated (0.67×). Transcriptome and proteome data very often do not overlap [36]. On the other hand, there are studies where proteome and previously published transcriptome data do align; however, this is in an experimental setting involving only diploids [37].

There is a lack of data where triploid and tetraploid Arabidopsis are compared side by side to their diploid counterparts under various stressors. Therefore, in this study, we wanted to comparatively quantify the effect of two stressors, elevated temperature and salinity, on the 2n, 3n, and 4n ploidy series in Arabidopsis at the proteomic level in order to describe novel differentially expressed proteins. Also, we used chlorophyll fluorescence as a stress resilience measure among the tested cytotypes.

## 2. Materials and Methods

### 2.1. Plant Material

In the study, we utilized the Col accession of Arabidopsis, with three levels of ploidy (2n, 3n, and 4n). Alongside naturally diploid individuals, tetraploid plants were created de novo by treating diploid specimens with the cytostatic agent colchicine. A solution of 0.25% (*w*/*v*) colchicine and 0.2% of the non-ionic surfactant Silwet L-77 were applied directly to the apical meristem of diploid plants (20 μL) [38]. Following self-pollination, seeds from these plants were collected and their ploidy was tested in two ways: by chromosome count and by a triploid block test. To confirm the ploidy level by chromosome count, chromosomal staining of pollen mother cells in prophase I of meiosis was performed using the fluorescent dye DAPI (Sigma-Aldrich, Darmstadt, Germany). Immature flower buds approximately 0.5 mm in size were collected and fixed in a solution of 96% ethanol and glacial acetic acid in a 3:1 ratio for two hours. The fixed buds were subsequently stored in 70% ethanol until slide preparation. Prior to staining, buds were rinsed on a microscope slide with sterile water and cold (4 °C) 0.1 M citrate buffer (pH 4.7) twice for five minutes each. Enzymatic digestion of the cell wall was performed by applying 10 μL of enzyme solution (0.42% pectolyase, 0.42% cellulase, and 0.42% driselase; all *w*/*v*) in a humid chamber for 35 min at 37 °C. The reaction was terminated by removing the enzyme with filter paper and adding 0.1 M citrate buffer (pH 4.7). Subsequently, 5 μL of 60% acetic acid was added, and the tissue was gently macerated using a histological needle. A coverslip was placed over the sample, and light thumb pressure was applied to squash and spread the tissue. The preparation was then dehydrated by freezing with carbon dioxide. After removing the coverslip, the slide was allowed to air dry. DAPI staining was performed by applying 200 μL of DAPI solution (2 µg mL^−1^), after which a new coverslip was placed, and the slide was incubated for 20 min in the dark at room temperature. Following removal of the coverslip, preparations were mounted using Dako Fluorescence Mounting Medium (Dako North America Inc., Carpinteria, CA, USA) and stored at 4 °C overnight. Microscopic observation was conducted using an Olympus BX51 fluorescence microscope equipped with a blue filter and imaged with a CCD camera (Olympus DP70). Immersion objectives were used for visualization, and the total magnification was 1000×.

In addition to chromosome counts, the ploidy of 4n candidate plants was further tested by using the triploid block test, a well-documented postzygotic reproductive barrier in Arabidopsis where the progeny of a 2n (female) × 4n (male) cross displays a strong seed abortion phenotype [39,40]. When we crossed a diploid female parent to a true tetraploid candidate male parent, the majority of seeds were aborted (due to maternal to paternal imbalance of the endosperm ploidy). On the other hand, the 4n (female) × 2n (male) reciprocal cross gave viable and normal looking seeds, like a standard diploid to diploid cross. Tetraploid plants screened by chromosome counts and a triploid block cross were self-pollinated for two generations prior to the beginning of the experiment.

Triploid plants were generated through an interploidy cross, by manually applying pollen from diploid male parent to the stigmas of tetraploid female parent. The ploidy was tested by chromosome counting of pollen mother cells as previously described for tetraploids. To phenotypically corroborate the determined cytotype of triploids, they were allowed to set seeds. Seeds from candidate triploids were very heterogeneous in shape and size, giving rise to phenotypically versatile plants, some of which were semi-sterile or sterile, all being hallmarks of triploid offspring [8].

### 2.2. Growth Conditions

For the stress experiment, seeds of all three ploidy levels (2n, 3n, and 4n) were surface-sterilized using 50% sodium hypochlorite and sowed on Murashige and Skoog Basal Medium (MS; Merck, Darmstadt, Germany) supplemented with 1% sucrose (Merck, Darmstadt, Germany). After overnight stratification at 4 °C, the seeds were transferred to the climate chamber and maintained under short-day conditions (8 h light/16 h dark) at 22 °C, 50% relative humidity and an illumination level of 130 µmol photons m^−2^ s^−1^ in the Fitoclima D1200 growth chamber (Aralab, Sintra, Portugal). Two weeks post germination, seedlings were transplanted from the MS medium to the soil substrate (Steckmedium, Klasmann, Geeste, Germany) and grown for approximately two months under the same conditions before heat or salt stress was applied. This growth regime was applied in order to suppress flowering and robust rosette development. Forty-eight plants were grown per ploidy level.

### 2.3. Experimental Stress Conditions and Detection of Stress Response

#### 2.3.1. Heat Stress

Twenty-four plants of each examined ploidy level (2n, 3n, 4n) were exposed to heat stress. Plants were grown and stressed in a plant growth chamber (Aralab, Sintra, Portugal) at 45 °C and 50% humidity for 3 h. Another 24 plants of each ploidy level were used as controls, at 22 °C and 50% humidity. One moderately developed rosette leaf was taken from each plant immediately after stress and 72 h after stress, and these samples were used for proline concentration analysis. Seventy-two hours after stress, several mid-developed rosette leaves were collected from four different plants for proteome analysis.

#### 2.3.2. Salt Stress

Twenty-four plants of each examined ploidy level (2n, 3n, 4n) were watered for three days with 300 mM NaCl solution [12] at 22 °C and 50% humidity. As a control group, 24 untreated plants of each ploidy level were used. These plants were watered with tap water and grown under the same temperature and humidity conditions as the plants exposed to salt stress. One moderately developed rosette leaf was taken from each plant 24 and 48 h after stress exposure, and these samples were used for proline concentration analysis. Several mid-developed rosette leaves were taken from four different plants 48 h after stress and used in proteome analysis.

### 2.4. Measuring of Chlorophyll Fluorescence

Chlorophyll *a* polyphasic fluorescence was measured using the OJIP test with a FluorPen FP100 fluorometer (Photon Systems Instruments, Drásov, Czech Republic). For each ploidy level, four control plants and four stressed plants (either heat- or salt-stressed) were assessed. Measurements were conducted on two mid-developed rosette leaves per plant. Chlorophyll *a* fluorescence was measured on mature leaves either immediately or 72 h after heat stress exposure, and at 24 and 48 h following salt stress. Prior to measurement, plants were dark-adapted for 30 min to halt photosynthesis and oxidize plastoquinone. The OJIP test monitors chlorophyll fluorescence induction during the dark-to-light transition, where fluorescence increases from the minimal level (*F*_0_) to the maximum (*F*_m_) under saturating light pulses [41]. The difference between *F*_m_ and *F*_0_ represents variable fluorescence (*F*_v_), and the *F*_v_/*F*_m_ ratio is a widely used parameter that reflects the maximum photochemical efficiency of photosystem II, providing insight into the degree of stress experienced by the plant: *F*_v_/*F*_m_ = (*F*_m_ − *F*_0_)/*F*_m_ [42]. Additionally, the performance index (*PI*_ABS_), which quantifies the overall efficiency of electron transport through photosystem II, was calculated [43]. Both *F*_v_/*F*_m_ and *PI*_ABS_ values were analyzed and graphically represented in relation to ploidy level and sampling time following stress exposure. These measurements served to confirm that the applied conditions effectively induced plant stress in control plants and comparatively measured the degree of stress in polyploids.

### 2.5. Measurement of Concentration of Proline

Proline extraction and quantification were performed following the standard protocol [44]. For each ploidy level, samples were taken from four plants subjected to either heat or salt stress, along with four control plants. Sampling was performed immediately and 72 h after heat stress, and at 24 and 48 h post salt stress. Each sample consisted of one rosette leaf, with an approximate fresh mass of 50 mg. This kind of sampling was performed for both heat and salt stress. Leaf tissue was ground in a mortar with liquid nitrogen to a fine powder, followed by the addition of 1 mL of 70% ethanol. Samples were centrifuged at 10,000× *g* for 10 min using an Eppendorf Centrifuge 5804R, and the supernatant was collected. Proline standards (0.05, 0.1, 0.25, 0.5, and 1 mM) were prepared by dissolving proline in 70% ethanol. In total, 1 mL of reaction mixture (1% ninhydrin *w*/*v*, 60% acetic acid *v*/*v*, and 20% ethanol *v*/*v*) was added to 100 μL of each extract or standard. The mixture was incubated at 95 °C for 20 min. Absorbance was measured at 520 nm using a Specord 50 PLUS spectrophotometer (Analytik Jena AG, Jena, Germany) with 1 cm path length cuvettes. The reaction mixture served as the blank. A standard calibration curve was designed using the known proline standards. Proline concentrations in the extract (mM) were calculated and expressed per milligram of fresh tissue (nmol/mg) using the following formula: proline (nmol mg^−1^) = [c (Proline, mM) × 1.1 mL]/mg of sample.

### 2.6. Extraction of Total Proteins from Leaves and Analysis

Proteins were extracted from plant material collected 72 h after heat stress and 48 h after salt stress treatment, which had been frozen at −80 °C. One biological replica with five leaves was used for every sample. For 2D electrophoresis, the phenol extraction protocol was performed according to a published procedure [41]. Protein concentration was determined by the modified Bradford method using a UV/Vis spectrophotometer UV-4 (Unicam, Cambridge, UK) and bovine serum albumin (BSA) as a standard [45].

The first dimension, isoelectric focusing (IEF), was performed using 13 cm long strips (pH 3–10) by Immobiline DryStrip Gels (Cytiva, Marlborough, MA, USA) for samples of temperature stress, and 18 cm long, immobilized pH gradient (IPG) strips, pH 4–7, for salt stress, in the IPGphor system (GE Healthcare, Chicago, IL, USA), according to [46]. The IPG strips were stored at −80 °C until use. IPG strips were thawed and incubated for 15 min in a buffer composed of 0.05 M Tris–HCl pH 8.8, 6 M urea, and 2% SDS (*w*/*v*) containing 130 mM dithiothreitol (DTT) and then for 15 min in a buffer of the same composition, but with 135 mM iodoacetamide instead of DTT. The second dimension was performed by sodium dodecyl sulfate–polyacrylamide gel electrophoresis (SDS-PAGE) as described in [47], using the PROTEAN II xi system (BioRad, Hercules, CA, USA). The 12% gel (12% T, 2.67% C) dimensions were 16 × 16 cm size and 0.75 mm thickness. Equilibrated IPG strips were laid on top of the gel, and agarose (γ = 5 g dm^−3^) was cast on top to hold the strips in place. Electrophoresis was performed at 100 V for 30 min and then at 220 V until the bromophenol blue ran off the gel. Upon completion, the gels were removed and stained with Coomassie Brilliant Blue (Thermo Fisher Scientific, Waltham, MA, USA) and destained with destaining solution. After destaining, the gels were scanned using an Epson Perfection V700 Photo, stored in the destaining solution, and refrigerated at 4 °C until further sampling.

### 2.7. Database Search, Comparative Analysis of Scanned Gels and Mass Spectrometry

By examining the interactive map of the polyacrylamide gel obtained through 2D electrophoresis of proteins from green tissue of Arabidopsis at the web address “https://gelmap.de/49# (accessed on 20 November 2024)” and using the SWISS-2DPAGE database from the Expasy server, we determined the approximate positions of stress-related proteins. Comparative analysis of scanned gels using the ImageMaster 2D Platinum 7.0 (GE Healthcare Life Sciences, Marlborough, MA, USA) software identified protein spots with the highest relative differences in intensity between control and treated samples. A total of eight samples were selected for mass spectrometry analysis—four for salt stress and four for heat stress—taken from the above-mentioned gels.

The gel slices were destained with solution of 5 mM NH_4_HCO_3_ and 50% acetonitrile, followed by dehydration of the gel slice with 100% acetonitrile. Disulfide bonds were reduced with 10 mM DTT in 20 mM NH_4_HCO_3_ for 45 min at 56 °C, and alkylated with iodoacetamide (27.5 mM, 30 min). Excess reagents were removed by several washes with 5 mM NH_4_HCO_3_ and 50% acetonitrile, while the gel slice was dehydrated with 100% acetonitrile and dried in SpeedVac. The gel slice was reswollen in 20 mM NH_4_HCO_3_ containing 12.5 ng/µL trypsin (Promega, Madison, WI, USA). The proteins were digested in-gel overnight at 37 °C, and digested peptides were extracted with 50% acetonitrile, 1% TFA and 80% acetonitrile, and 1% TFA, pooled, concentrated by evaporation, and desalted on stage tips. LC-MS/MS analysis was performed using the EASY-nLC™ 1200 System (Thermo Fisher Scientific) coupled with a Q Exactive Plus mass spectrometer (Thermo Fisher Scientific). The raw data were processed with Proteome Discoverer 2.4 (Thermo Fisher Scientific), by searching against the Arabidopsis protein database.

## 3. Results

### 3.1. Confirmation of the Ploidy Level of Plant Material

**Figure 1:**
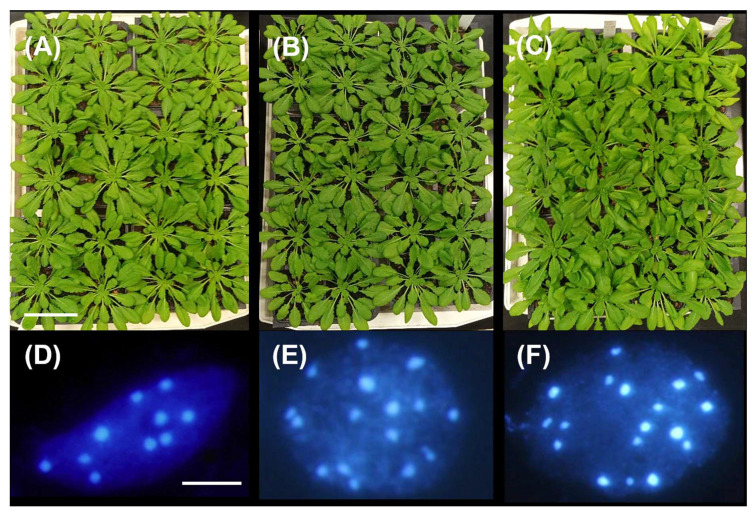
Diploid (**A**), triploid (**B**), and tetraploid (**C**) Arabidopsis plants at 60 DAG, grown in short-day conditions (8 h light/16 h dark) at the beginning of the stress treatments. DAPI- stained interphase nuclei of a pollen mother cell (PMC) of 2n (**D**), 3n (**E**), and 4n (**F**) show strong staining of chromocenters, which represent the corresponding chromosome numbers: 2n (2x = 10), 3n (3x = 15), and 4n (4x = 20). Scale bar: 5 cm (**A**) and 5 µm (**B**).

The cytotype of our 2n, 3n, and 4n plants was confirmed prior to the beginning of the experiment. The rosette phenotype of diploid and triploid plant at 60 DAG was rather similar (Figure 1A,B). On the other hand, tetraploids had a visibly larger rosette (Figure 1C). At the cellular level, DAPI staining revealed AT-rich centromeric and pericentromeric heterochromatic regions, i.e., the chromocenters. In interphase nuclei, the number of chromocenters usually corresponds to the expected number of chromosomes (Figure 1D–F).

### 3.2. Chlorophyll Fluorescence as an Indicator of Stress

**Figure 2:**
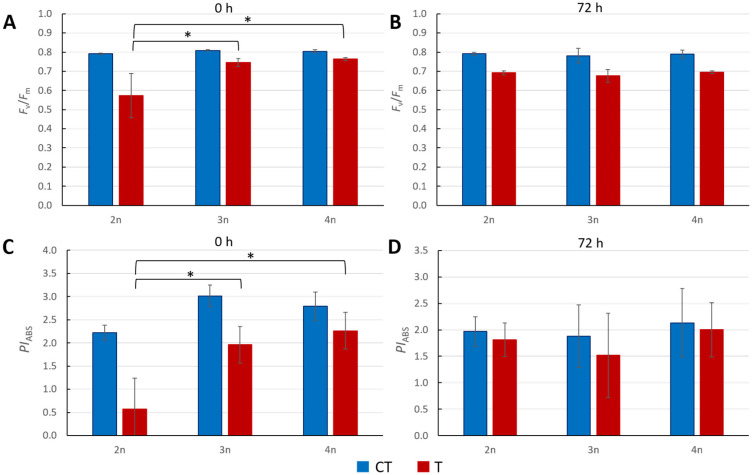
Heat stress at 45 °C for 3 h. (**A**,**B**) Maximum quantum yield of primary PSII photochemistry (*F*_v_/*F*_m_) at 0 h and 72 h after stress. (**C**,**D**) Performance index on absorption basis (*PI*_ABS_) at 0 h and 72 h post stress. Each column represents the mean value of eight biological replicas. The standard deviation is indicated on the columns. Significant difference was calculated using *t*-tests (*p* < 0.05), indicated with an asterisk (*). CT—control plants; T—treated plants.

Three cytotypes, 2n, 3n, and 4n, were subjected to temperature stress at 45 °C for 3 h, and the maximum quantum yield of primary PSII photochemistry (*F*_v_/*F*_m_) was measured immediately. Both polyploids showed statistically higher *F*_v_/*F*_m_ values compared to control plants (Figure 2A). No statistically significant differences were observed 72 h after stress exposure between treated polyploids and treated diploids.

Measurements of the performance index on absorption basis (*PI*_ABS_) immediately after stress confirmed our previous observation, showing significant differences between diploid and triploid plants, as well as diploid and tetraploid plants (Figure 2C). At 72 h heat stress post treatment, there were no significant *PI*_ABS_ differences between diploids and polyploids.

**Figure 3:**
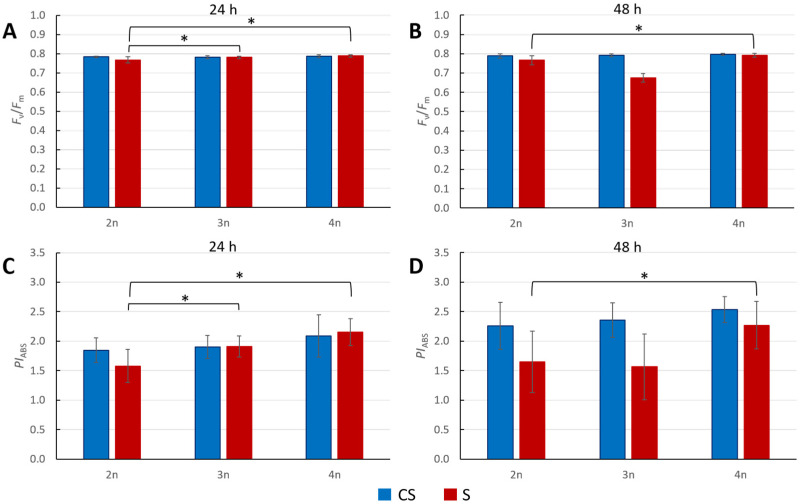
Salt stress with 300 mM NaCl. (**A**,**B**) Maximum quantum yield of primary PSII photochemistry (*F*_v_/*F*_m_) at 24 h and 48 h post stress. (**C**,**D**) Performance index on absorption basis (*PI*_ABS_) at 24 h and 48 h after stress. Each column represents the mean value of eight biological replicates. The standard deviation is indicated on the columns; statistically significant difference was calculated using *t*-tests (*p* < 0.05), indicated with an asterisk (*). CS—control plants; S—treated plants.

We also measured the photosynthetic reaction of plants on salt stress. After 24 h of salt treatment, *F*_v_/*F*_m_ values of diploid plants were statically lower than the ones in 3n and 4n plants (Figure 3A). However, the measured values of *F*_v_/*F*_m_ 48 h after exposure to stress showed a statistically significant difference only between control and treated diploid plants, while in the case of polyploids, the difference between control and treated plants was not statistically significant (Figure 3B).

Measurements of *PI*_ABS_ in plants after 24 h of salt stress showed a similar trend. Triploids and tetraploids had statistically higher values compared to diploids. (Figure 3C). The next measurement was after 48 h of salt stress. The only significant difference was observed between 2n and 4n plants. (Figure 3D). For both measurements, *F*_v_/*F*_m_ and *PI*_ABS_, triploids reacted similarly to 2n controls.

### 3.3. Concentration of Proline as Stress Indication

**Figure 4:**
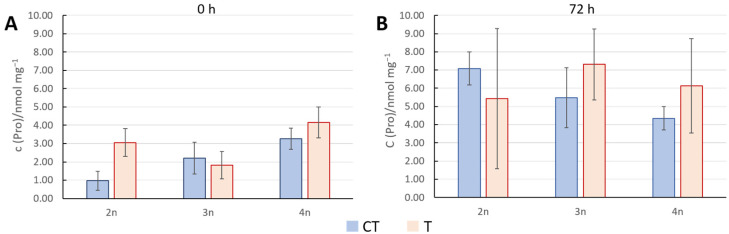
Proline concentration upon heat stress. Proline concentration in Arabidopsis plants of different ploidy levels measured immediately (**A**) and 72 h (**B**) after exposure to heat stress at 45 °C for 3 h, along with corresponding values for control plants. Each column represents the mean value of four biological replicates. The standard deviation is indicated on the columns. No statistical difference was detected between diploids and polyploids under stress by the *t*-test (*p* < 0.05). CT—control plants; T—treated plants.

Proline concentration was measured from leaf samples taken immediately or 72 h after exposure to 45 °C for 3 h. Measured proline concentration immediately after stress showed no statistically significant difference when comparing diploids to each polyploid (Figure 4A). The only significant difference was between triploids and tetraploids, where triploids turned out to be less sensitive to heat stress than tetraploids.

**Figure 5:**
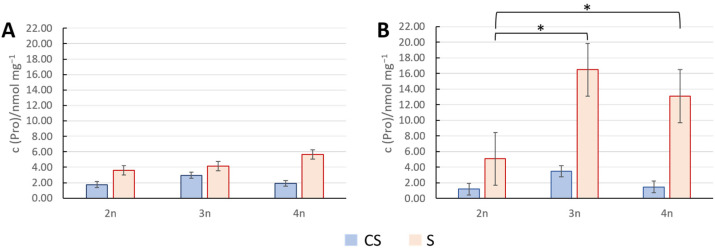
Proline concentration upon stress with 300 mM NaCl. Concentration of proline in Arabidopsis plants with different ploidy levels, measured 24 h (**A**) and 48 h (**B**) after exposure. Each column represents the mean value of four biological replicates. The standard deviation is indicated on the columns; statistically significant difference was calculated by using the *t*-test (*p* < 0.05), indicated with an asterisk (*). CS—control plants; S—treated plants.

Further, comparing proline concentrations between diploids and polyploids 72 h after temperature stress revealed a large spread of measured values with no significant differences. Then, we tested proline salt stress responses in 2n, 3n, and 4n plants at 24 and 48 h after stress application (Figure 5). By comparing proline concentration in treated diploids and treated polyploids 24 h after exposure to stress, a difference was observed between 2n vs. 3n and 2n vs. 4n (Figure 5A), indicating a slightly higher stress resilience in polyploids. In addition, after 48 h, this trend continued. Both polyploids exerted higher proline accumulation in comparison to diploids (Figure 5B).

### 3.4. 2D Electrophoresis in Polyacrylamide Gel, Computer Analysis and Identification by Mass Spectrometry

**Figure 6:**
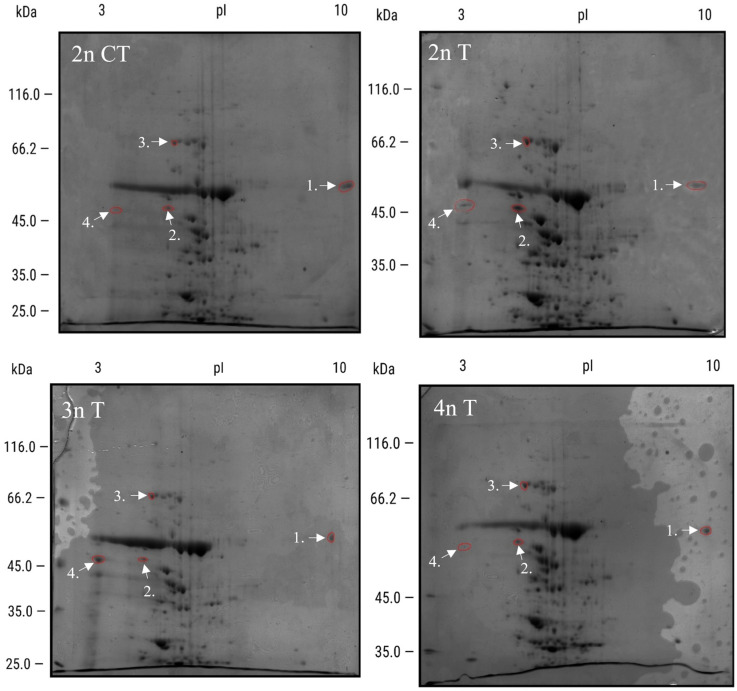
Expression patterns of cellular proteins from Arabidopsis plants exposed to heat stress at 45 °C for 3 h, taken after 72 h after exposure to stress, obtained by two-dimensional electrophoresis (2-DE). In the first dimension (IEF), 300 μg of proteins were resolved in 13 cm IPG strips with a pH gradient of 3–10. In the second dimension, proteins were separated on a 12% SDS polyacrylamide gel and then stained with Coomassie Brilliant Blue. Samples showing the strongest differential expression are labeled 1–4 and were identified using MALDI-TOF/TOF mass spectrometry. CT—control plants; T—heat-treated plant.

**Figure 7:**
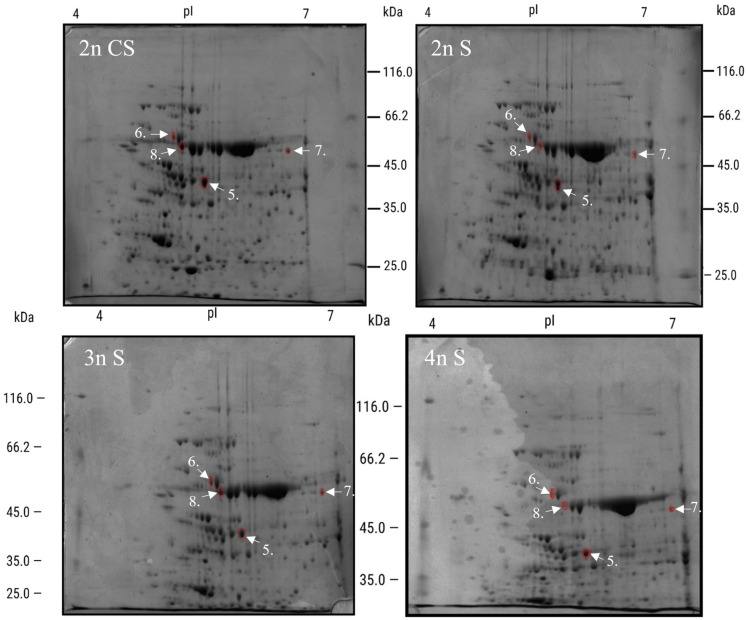
Expression patterns of cellular proteins from diploid and polyploid plant samples treated for 48 h with 300 mM NaCl and the corresponding 2n control obtained by as two-dimensional electrophoresis (2-DE). In the first dimension (IEF), 300 μg of proteins were resolved in 18 cm IPG strips with a pH gradient of 4–7. In the second dimension, proteins were separated on a 12% SDS polyacrylamide gel and then stained with Coomassie Brilliant Blue. Samples showing the strongest differential expression are labeled 5–8 and were identified using MALDI-TOF/TOF mass spectrometry. CS—control plant; S—salt-treated plant.

To better understand the influence of plant polyploidy on stress responses, we measured changes in protein expression using 2D SDS-PAGE. Two-dimensional protein electrophoresis was performed on three different cytotypes (2n, 3n, and 4n) subjected to either heat (Figure 6) or salt stress (Figure 7).

Proteins visible on all gels (Figure 6 and Figure 7) using ImageMaster 2D Platinum were analyzed and compared to control gels. Those with the highest differences within each ploidy compared to controls are presented in Table 1.

The first group of spots was from heat stress: Treated protein 1 had similarly lower expression in 2n and 3n, but in 4n, it had 26.71% higher expression. Spot 2 had different expression across all n plants—highest in 3n, lowest in 4n, and 100% higher in 2n. Protein 3 had similar 2n and 4n value, but was downregulated in 3n, while the last spot measured was highest in 3n and lowest in 4n.

The next group of proteins was chosen from salt stress: Spot 5 had the highest level in 2n, at 30% higher, was 30% lower in 3n, and similar to the control in 4n. In spot 6, 2n showed no difference from the control, while 3n-treated protein was upregulated by 136%, and 4n was downregulated by 30.18%. At spot 7, there was no difference in 2n and 4n, while in 3n, the protein was 23.83% downregulated compared to the control. For the last protein, 2n was downregulated by 59.18% and 4n by 11.41%, while 3n was 80.15% upregulated.

To have a better overview, Table 2 presents a comparison restricted to proteins from treated 2nT, 3nT, and 4nT samples, excluding the control proteins derived from 2D gels as shown in Table 1.

In the heat stress part of Table 2, at protein 1, 2n and 3n were similar, while 4n had 126% higher expression. Protein 2 had lower expression in 3n and 4n plants compared to 2n. Protein three had similar expression in 2n and 4n, and lower expression in 3n. Protein 4 had the lowest expression in 4n but 165% increased expression in 3n compared to 2n.

After salt stress, protein 5 had similar expression in 2n and 4n, but 21% lower expression in 3n. Protein 6 had increased expression in 3n (13%) and 4n (38%) compared to 2n. In protein 7, 2n and 4n had similar expression, while 3n was increased (23%). For protein 8, 2n and 4n had the same expression, while 3n was increased (71%).

Finally, we have isolated selected differentially expressed proteins (1.–8.) and identified them by mass spectrometry (Table 3). The most abundant protein in each sample (spot) was identified by comparing the abundance, PSMs (Peptide Spectrum Matches), and Sequest Score parameters, and selecting peptides with significantly higher values. Detailed values for PSMs and Sequest Scores for each sample are provided in the (Table 3). In some spots, there were two proteins of the same size and similar p*I* values, but lower PSM values (not mentioned in Table 3).

The most differentially expressed proteins we identified (1, 3, 4, 5, and 6) were from the chloroplast (Table 3). Other proteins were from nuclear or cytosolic-like dehydrin COR47 (2), or peroxisome glutamate--glyoxylate aminotransferase 1 and 2 (7).

In 2n plants, heat stress reduced the large chain of ribulose bisphosphate carboxylase (1; Table 3) and increased the quantity of dehydrin COR47 (2), heat shock 70 kDa protein 6, chloroplastic (3), and, slightly, chloroplastic ribulose bisphosphate carboxylase/oxygenase activase (4). For all mentioned proteins, 3n Arabidopsis had a similar trend to 2n (1–4), while 4n had a different heat response: it showed an Increase in proteins 1 and 3 and a downregulation of proteins 2 and 4.

Then we measured the differences after salt stress and selected four proteins (5–8; Table 1). In 2n plants, we found an increase in three proteins: Ribulose bisphosphate carboxylase/oxygenase activase, chloroplastic (5), ATP synthase subunit alpha, chloroplastic (6), which increased slightly, and glutamate-glyoxylate aminotransferase 1 with 2 (7). On the other hand, protein ribulose bisphosphate carboxylase large chain (8) was downregulated. Triploid and tetraploid plants reacted differently. In 3n plants, proteins (5) and (7) were downregulated, whereas (6) and (8) were upregulated. In 4n plants, protein levels were somewhat different compared to that of 3n: proteins (5) and (7) had the same expression as controls, while (6) and (8) had lower expression.

## 4. Discussion

Although many studies have described the effect of either heat or salt stress on model or crop plants, relatively few studies have addressed the specific differences in stress responses across a polyploid series of Arabidopsis. For instance, the salt stress tolerance of autotetraploids was studied previously, but limited to plant survival and reproductive success under stress, without examining the expression of stress-related proteins [30]. Also, the effect of heat and salt stress was compared in different Arabidopsis accessions, but only in diploids [48]. Similarly, a polyploid series of 2n, 4n, 6n, and 8n Arabidopsis was thoroughly compared, but only in non-stress conditions [27]. To our knowledge, there are no other studies investigating photosynthesis-related responses and differential proteome expression of 2n, 3n, and 4n Arabidopsis cytotypes upon heat and salt stress.

It is well known that the photosynthetic apparatus is among the most sensitive to stress conditions, which can damage the chloroplast membrane, photosystem II, RUBISCO activase, and cyclic electron transport [15,49,50]. These changes decrease the maximum fluorescence intensity (*F*_m_) and increase the minimal fluorescence (*F*_0_), eventually causing a drop in maximum photochemical efficiency (*F*_v_/*F*_m_) and the absolute performance index (*PI*_ABS_), which approximates to the stress level of the photosynthesis apparatus.

Immediately after exposure of 2n, 3n, and 4n plants to heat stress (45 °C, 3 h), we observed that all cytotypes showed a decrease in photochemical efficiency (*F*_v_/*F*_m_) when compared to the corresponding untreated cytotype (Figure 2A), indicating that plants indeed experience stress under our experimental conditions. Absolute performance index (*PI*_ABS_) values also decreased between controls and treated plants at each ploidy level (Figure 2C). Further, when comparing heat-exposed 2n plants with 3n and 4n plants immediately after the stress, we observed that the photosynthetic apparatus of 3n and 4n plants was significantly less affected (Figure 2A,C), as measured by both *F*_v_/*F*_m_ as well as *PI*_ABS_ values. The significantly higher values in high heat-exposed triploid and tetraploid plants, compared to diploids, indicate greater resilience of both triploids and tetraploids under heat stress (Figure 2A,C). A similar observation showed greater tolerance of tetraploid Arabidopsis under drought conditions [51], but did not include triploids. Also, another study showed that heat response in Arabidopsis can already be detected 5 min after stress induction but gradually declines to normal within 3 h of recovery [52,53].

In line with this observation, we also found that a recovery phase, after heat stress, diminishes any significant difference in *F*_v_/*F*_m_ or *PI*_ABS_ values between treated and untreated plants, irrespective of their ploidy (Figure 2B,D).

Furthermore, *F*_v_/*F*_m_ and *PI*_ABS_ photosynthetic parameters in plants exposed to salt stress (300 mM NaCl) for 24 h showed statistically significant differences between control and treated plants only in diploids (Figure 3A,C). This suggests that the photosynthetic apparatus of diploids was significantly affected by salt stress, while polyploids showed more resilience (Figure 3). A statistically significant difference in *F*_v_/*F*_m_ and *PI*_ABS_ values was also found between treated diploids and polyploids, confirming previous findings [32,54] that tetraploid Arabidopsis cope better with salt stress than diploids. In our study, next to tetraploids, triploids also showed a higher salt stress tolerance.

After 48 h of salt stress, *F*_v_/*F*_m_ and still *PI*_ABS_ values again showed significant tolerance in treated 4n plants in relation to 2n controls (Figure 3B), while triploids seemed to be as sensitive as diploids. Here, for the first time, we observed that triploids react differently than tetraploids upon the applied stress. It seems that triploids, in this case, exert a cytotype-specific stress response based on a possible change in stress perception, which could be a consequence of regulatory challenges derived from triploidy itself. The exact same observation was obtained when *PI*_ABS_ was measured: tetraploids showed greater tolerance, and triploids behaved like diploid plants under salt stress (Figure 3D).

An increase in free proline synthesis is a physiological response to stress. Its importance lies in its roles as an osmolyte, chaperone, pH regulator, and scavenger of ROS [55]. Monitoring proline levels as a stress indicator is a common practice [56,57,58,59], but to our knowledge, no studies have examined the correlation between proline levels, stress, and polyploidy.

Our proline level measurements after heat and salt tests showed diverse results (Figure 4 and Figure 5). A significant difference in proline concentration between control and treated plants immediately after 24 h heat stress was observed only in diploids (Figure 4A), which indicated that diploids perceived and reacted to heat stress by upregulating proline concentrations. Triploids and tetraploids, on the other hand, seem to react slower due to altered stress perception or signaling (Figure 4A). This could be in line with some previous findings, pointing to increased proline catabolism in polyploids as a heat stress-coping mechanism [60]. Alternatively, a study found that heat stress leads to the accumulation of transcripts encoding P5CR, a key enzyme in proline synthesis, but does not result in immediate proline accumulation [61]. Our 72 h post-heat stress proline measurement was highly dissipated, making this dataset rather inconclusive, at least for this data point (Figure 4B).

Further, 24 h after salt stress, proline concentrations were significantly different between controls and treated plants in both diploids and tetraploids (Figure 5A). In triploids, there was a visible increasing trend in proline concentrations, though not statistically significant (Figure 5A). Forty-eight hours after salt exposure, proline concentrations in treated plants were significantly higher than in all controls (Figure 5B), confirming that plants experienced stress, corroborating previous findings that proline accumulates under stress conditions [55]. A statistically significant difference in proline concentration was also observed between diploids and each polyploid (Figure 5B), with polyploids showing two- to three-fold higher proline concentrations. This suggests that polyploids demonstrate better salt stress tolerance, as higher proline levels implicate improved stress defense capacity, as shown by [62]. In line with this finding, it was shown that exogenous proline treatment under stress improves cell growth, biomass, photosynthetic activity, and seed germination across many plant species [63]. To conclude, triploid and tetraploid plants might have a higher capacity for proline accumulation, reflecting their superior tolerance to salt stress in our experimental settings.

We have analyzed quantities and identified selected proteins by mass spectrometry from 2D SDS-PAGE gels. Some of the identified plant proteins are well known for being variable under temperature stress conditions [64,65,66]. Furthermore, we detected differences between observed and theoretical Mw and p*I* values that are likely attributable to posttranslational modifications and different posttranscriptional cleavages [65].

In Table 1, we show ratios of protein spot volumes of samples exposed to temperature or salt stress and corresponding controls. We observed significant difference in expression for several proteins, as follows:

In diploids, the protein identified in spot 1 was ribulose bisphosphate carboxylase large chain (RUBISCO) and is significantly downregulated under heat stress conditions compared to the control (Table 1), which is a trend reported previously [65]. Triploids also showed RUBISCO downregulation under heat stress, while in tetraploids, RUBISCO was upregulated. Similar results are also visible in Table 2. A difference in expression during heat stress was observed: downregulation of RUBISCO occurred in Arabidopsis [65] and rice [67], while upregulation of RUBISCO was recorded in rice and wheat [68]. In rice seedlings, the upregulation of RUBISCO was a way to maintain CO_2_ fixation during such stress [69].

Sample 2 was identified as dehydrin COR47 (Table 3), which is part of a drought response mechanism, as well as temperature (heat/cold) and osmotic stress response [70]. Dehydrins generally play a role in preventing dehydration and oxidative damage, and due to their chaperone activity, they help prevent the degradation of cellular proteins under stress [64]. In diploids and triploids, higher expression of dehydrin COR47 was observed under stress conditions, compared to corresponding 2n and 3n controls. On the other hand, in stressed tetraploids, dehydrin COR47 was reduced compared to corresponding 4n controls (Table 1). However, comparing stressed diploids and stressed polyploids (Table 2) showed lower expression of dehydrin COR47 in polyploids. One of the reasons for that might be lower stress levels in polyploid plants, which is in accordance with results from the OJIP test. In other plants, like wheat [71] and sunflower [72], variations in dehydrin accumulation patterns have been observed under drought stress, depending on the plant organ and developmental stage.

In sample 3 (Table 3), chloroplastic heat shock 70 kDa protein 6 and 7 reacted to temperature stress. Some of their functions include acting as chaperones, preventing protein aggregation, and mediating their correct localization within the cell [65,73,74]. They are not only important for stress response, but their chaperone roles perform an important job in plant response to any changes in homeostasis [75]. In our study, we have shown that their response depends on inherent changes associated with genome polyploidization, in which their chaperone role could be of great importance in relation to changes in the regulation of stress-responsive gene expression.

Sample 4 (Table 3) was identified as ribulose bisphosphate carboxylase/oxygenase activase, chloroplastic (RCA). RCA is a very temperature-sensitive enzyme [16] that degrades even at moderate heat stress (35 °C). Its expression is closely linked to the proper functioning of the photosynthetic apparatus, as it functions as a chaperone, essential for the function of RUBISCO [76]. According to [71,77], the amplification of RCA in rice (*Oryza sativa*) leads to better heat stress tolerance. Also, transgenic Arabidopsis plants with temperature-resistant RCA show better resistance to heat stress [78]. In our study, stressed diploid and triploid plants show higher expression of RCA compared to 2n and 3n control plants, while tetraploids show a decrease in expression (Table 1). Treated triploid plants showed higher expression compared to diploid plants, while tetraploid ones had lower level of expression (Table 2). These results imply different perception of stress between triploid and tetraploid plants.

After salt stress, we identified the same four proteins as after heat stress (Table 3, proteins 5–8). In sample 5, the protein isolated was RCA. According to [79], in times of salt stress, its expression is reduced in order to reduce the activity of RUBISCO and, consequently, the entire Calvin cycle. Comparing treated and control plants (Table 1, protein 5), an increased expression level was observed in diploids, a decreased expression level in triploids, and an unchanged expression level in tetraploids. Furthermore, both treated polyploid have somewhat smaller expressions of RUBISCO in relation to treated diploids (Table 2, protein 5), which indicates a greater effect of salt stress on protein expression.

Protein 6 (Table 3) was identified as α ATP synthase subunit. In diploids, there was no differential expression of α ATP synthase subunit in stressed compared to control 2n plants. However, in triploids, α ATP synthase subunit levels were significantly higher, while in tetraploids, the levels were lowered in stressed plants compared to corresponding 3n and 4n controls (Table 1, protein 6). When comparing stressed 2n plants to stressed polyploid plants, we observed a higher α ATP synthase subunit protein quantity (Table 2, protein 6). We propose that this increase might allow higher salt stress tolerance. Given their crucial role in plant metabolism and energy production, these higher expression levels may be associated with greater salt stress tolerance in plants under stress [66].

The protein 7 spot was identified by mass spectrometry as two types of glutamate-glyoxylate aminotransferase, 1 and 2 (GGAT). It is located in the cell peroxisomes and is involved in amino acid content during photorespiration [77,80]. Mutants in the gene encoding glutamate-glyoxylate aminotransferase 1 show accumulation of H_2_O_2_, as a consequence of increased oxidative stress in plants [81]. The role of GGAT includes helping plants cope during stress response and changes in photosynthesis. During stress, 2n and 4n (Table 1) showed no difference in expression of GGAT, while in 3n, it was downregulated. Looking at protein values without controls (Table 2) shows that 2n and 4n have similar expression, much lower than 3n.

The protein isolated from spot 8 was the same as the one identified in spot 1: ribulose bisphosphate carboxylase large chain. In Arabidopsis [66], RUBISCO was less expressed compared to untreated controls under salt stress. In our study, diploids also showed downregulation of RUBISCO compared to 2n controls. Also, there was increased expression in triploids and decreased expression in tetraploids compared to the corresponding 3n and 4n untreated controls (Table 1, protein 8). However, comparing stressed 2n plants to stressed 3n plants, we measured an increase in expression levels. Further, stressed 4n plants had an equal expression as the stressed diploids. Such results suggest higher resistance of polyploid plants to salt stress, at least in triploids, which is in line with our observation of photosynthesis efficiency (Figure 2 and Figure 3) and proline levels (Figure 4 and Figure 5).

In conclusion, our results support the assumption that polyploid variants of Arabidopsis might be more resistant to heat and salt stress. Our results give further support to existing lines of evidence [1,32] that claim that polyploidy might be an important mechanism for adaptation of plants to unfavorable conditions. On a broader level, we can conclude that targeted polyploidization of certain commercially and nutritionally important species could represent a potentially significant strategy for addressing emerging abiotic stressors.

## Figures and Tables

**Table 1:** Spot volume ratio of proteins from plant samples exposed to heat stress of 45 °C for 3 h and corresponding controls, and plant samples exposed to salt stress and corresponding controls watered with pure H_2_O.

Protein Number from Gel	Ration of Volumes 2nT/2nCT	Ration of Volumes 3nT/3nCT	Ration of Volumes 4nT/4nCT
1.	0.3945	0.3012	1.2671
2.	2.4662	4.4988	0.7704
3.	2.1009	1.7031	2.0966
4.	1.2581	4.8661	0.3335
/	2nS/2nCS	3nS/3nCS	4nS/4nCS
5.	1.3085	0.7005	1.0339
6.	1.116	2.3662	0.6982
7.	1.1034	0.7617	1.0819
8.	0.4082	1.8015	0.8859

Definitions: 2n, 3n, and 4n—plant ploidy; T—plant exposed to heat stress; CT—untreated control plant for temperature stress; S—plants exposed to salt stress; CS—untreated control plants for salt stress.

**Table 2:** Quantitation of 2 D SDS-PAGE differential protein expression in diploid (2n), triploid (3n), and tetraploid (4n) plants for temperature (1.–4.) and salt (5.–8.) stress.

Sample No.	Spot Volume 3n T *	Relative Expression	Spot Volume 2n T	Relative Expression	Spot Volume 4n T
1.	21.1198	=	21.1871	↑	47.7674
2.	10.0316	↓	32.9035	↓	18.5064
3.	17.7087	↓	26.6486	↑	29.2735
4.	51.1399	↑	19.2608	↓	4.45263
/	3n S **	/	2n S	/	4n S
5.	51.2678	↓	65.2919	↓	60.628
6.	12.3193	↑	10.8297	↑	14.9568
7.	11.7888	↑	9.51844	=	9.95286
8.	24.6241	↑	14.3599	=	14.4623

* T—temperature 45 °C, 3 h; ** S—salt 300 mM NaCl. ↑—higher than 2n, ↓—lower than 2n, =—similar to 2n. Relative expression levels were assessed by comparing spot volumes from 3n and 4n samples relative to 2n samples.

**Table 3:** Identified proteins; the numbers are used in Table 1 and in 2D SDS-PAGE gels.

Prot. №	Protein Name	UniProt Accession	Theor. M_w_ (kDa)/pI	Seq. Cov. %	# PSMs
1., 8.	ribulose bisphosphate carboxylase large chain	O03042	52.9/5.88	58	334
2.	dehydrin COR47	P31168	29.9/4.74	40	24
3.	heat shock 70 kDa protein 6, chloroplastic	Q9STW6	76.5/5.07	36	127
3.	Heat shock 70 kDa protein 7, chloroplastic	Q9LTX9	76.99/5.17	31	96
4., 5.	ribulose bisphosphate carboxylase/oxygenase activase, chloroplastic	P10896	51.9/5.87	74	800
6.	ATP synthase subunit alpha, chloroplastic	P56757	55.3/5.19	37	147
7.	glutamate-glyoxylate aminotransferase 1	Q9LR30	53.3/6.49	63	194
	glutamate-glyoxylate aminotransferase 2	Q9S7E9	53.4/6.21	35	134

Multiple ordinal numbers next to one protein name indicate that the same protein was identified in >1 than sample. Theor. Mw—theoretical molecular weight, p*I*—isoelectric point, Seq. cov.—sequence coverage % (peptides), # PSMs—a number of Peptide Spectrum Matches.

## Data Availability

All data are available upon request to the corresponding authors.

## References

[1] Fawcett J.A., Maere S., Van De Peer Y. (2009). Plants with Double Genomes Might Have Had a Better Chance to Survive the Cretaceous—Tertiary Extinction Event. Proc. Natl. Acad. Sci. USA.

[2] Mangena P. (2023). Impact of Polyploidy Induction for Salinity Stress Mitigation in Soybean (*Glycine max* L. Merrill). Plants.

[3] Van De Peer Y., Mizrachi E., Marchal K. (2017). The Evolutionary Significance of Polyploidy. Nat. Rev. Genet..

[4] De Storme N., Geelen D. (2020). High Temperatures Alter Cross-over Distribution and Induce Male Meiotic Restitution in *Arabidopsis thaliana*. Commun. Biol..

[5] Lei X., Ning Y., Eid Elesawi I., Yang K., Chen C., Wang C., Liu B. (2020). Heat Stress Interferes with Chromosome Segregation and Cytokinesis during Male Meiosis in *Arabidopsis thaliana*. Plant Signal. Behav..

[6] Jin C., Sun L., Trinh H.K., Danny G. (2023). Heat Stress Promotes Haploid Formation during CENH3-Mediated Genome Elimination in *Arabidopsis*. Plant Reprod..

[7] Crhak Khaitova L., Mikulkova P., Pecinkova J., Kalidass M., Heckmann S., Lermontova I., Riha K. (2024). Heat Stress Impairs Centromere Structure and Segregation of Meiotic Chromosomes in *Arabidopsis*. Elife.

[8] Henry I.M., Dilkes B.P., Young K., Watson B., Wu H., Comai L. (2005). Aneuploidy and Genetic Variation in the *Arabidopsis thaliana* Triploid Response. Genetics.

[9] Zandalinas S.I., Peláez-Vico M.Á., Sinha R., Pascual L.S., Mittler R. (2024). The Impact of Multifactorial Stress Combination on Plants, Crops, and Ecosystems: How Should We Prepare for What Comes Next?. Plant J..

[10] Umezawa T., Mizuno K., Fujimura T. (2002). Discrimination of Genes Expressed in Response to the Ionic or Osmotic Effect of Salt Stress in Soybean with cDNA-AFLP. Plant Cell Environ..

[11] Zhu J.-K. (2016). Abiotic Stress Signaling and Responses in Plants. Cell.

[12] Yang Y.-B., Yin J., Huang L.-Q., Li J., Chen D.-K., Yao N. (2019). Salt Enhances Disease Resistance and Suppresses Cell Death in Ceramide Kinase Mutants. Plant Physiol..

[13] Singh A., Sharma M.K., Sengar R.S. (2017). Osmolytes: Proline Metabolism in Plants as Sensors of Abiotic Stress. J. Appl. Nat. Sci..

[14] Mansour M.M.F., Salama K.H.A., Hasanuzzaman M. (2020). Proline and Abiotic Stresses: Responses and Adaptation. Plant Ecophysiology and Adaptation under Climate Change: Mechanisms and Perspectives II: Mechanisms of Adaptation and Stress Amelioration.

[15] Allakhverdiev S.I., Kreslavski V.D., Klimov V.V., Los D.A., Carpentier R., Mohanty P. (2008). Heat Stress: An Overview of Molecular Responses in Photosynthesis. Photosynth. Res..

[16] Sharkey T.D., Zhang R. (2010). High Temperature Effects on Electron and Proton Circuits of Photosynthesis. J. Integr. Plant Biol..

[17] Lee J.H., Burdick L.H., Piatkowski B., Carrell A.A., Doktycz M.J., Pelletier D.A., Weston D.J. (2023). A Rapid Assay for Assessing Bacterial Effects on Arabidopsis Thermotolerance. Plant Methods.

[18] Xue H., Zhang F., Zhang Z.-H., Fu J.-F., Wang F., Zhang B., Ma Y. (2015). Differences in Salt Tolerance between Diploid and Autotetraploid Apple Seedlings Exposed to Salt Stress. Sci. Hortic..

[19] Tu Y., Jiang A., Gan L., Hossain M., Zhang J., Peng B., Xiong Y., Song Z., Cai D., Xu W. (2014). Genome Duplication Improves Rice Root Resistance to Salt Stress. Rice.

[20] Wang L., Cao S., Wang P., Lu K., Song Q., Zhao F.-J., Chen Z.J. (2021). DNA Hypomethylation in Tetraploid Rice Potentiates Stress-Responsive Gene Expression for Salt Tolerance. Proc. Natl. Acad. Sci. USA.

[21] Zhu H., Zhao S., Lu X., He N., Gao L., Dou J., Bie Z., Liu W. (2018). Genome Duplication Improves the Resistance of Watermelon Root to Salt Stress. Plant Physiol. Biochem..

[22] Shafieizargar A., Awang Y., Juraimi A.S., Othman R. (2013). Comparative Studies between Diploid and Tetraploid Dez Orange *Citrus sinensis* (L.) Osb. under Salinity Stress. Aust. J. Crop Sci..

[23] Podda A., Checcucci G., Mouhaya W., Centeno D., Rofidal V., Del Carratore R., Luro F., Morillon R., Ollitrault P., Maserti B.E. (2013). Salt-Stress Induced Changes in the Leaf Proteome of Diploid and Tetraploid Mandarins with Contrasting Na^+^ and Cl^−^ Accumulation Behaviour. J. Plant Physiol..

[24] Song X., Zhang M., Wang T., Duan Y., Ren J., Gao H., Fan Y., Xia Q., Cao H., Xie K. (2025). Polyploidization Leads to Salt Stress Resilience via Ethylene Signaling in Citrus Plants. New Phytol..

[25] Aycan M., Erkilic E.G., Ozgen Y., Poyraz I., Yildiz M. (2023). The Response of Sugar Beet (*Beta vulgaris* L.) Genotypes at Different Ploidy Levels to Salt (NaCl) Stress. Int. J. Plant Biol..

[26] Dong Y., Hu G., Yu J., Thu S.W., Grover C.E., Zhu S., Wendel J.F. (2020). Salt-tolerance Diversity in Diploid and Polyploid Cotton (*Gossypium*) Species. Plant J..

[27] Corneillie S., De Storme N., Van Acker R., Fangel J.U., De Bruyne M., De Rycke R., Geelen D., Willats W.G.T., Vanholme B., Boerjan W. (2019). Polyploidy Affects Plant Growth and Alters Cell Wall Composition. Plant Physiol..

[28] Pacey E.K., Maherali H., Husband B.C. (2022). Polyploidy Increases Storage but Decreases Structural Stability in *Arabidopsis thaliana*. Curr. Biol..

[29] Kumar A., Gupta N., Bahadur B., Venkat Rajam M., Sahijram L., Krishnamurthy K.V. (2015). Applications of Triploids in Agriculture. Plant Biology and Biotechnology: Volume II: Plant Genomics and Biotechnology.

[30] Tsai Y.-S., Liao Y.-C., Wei T.-Y., Yeh D.-M. (2024). Triploid Formation and Heat Tolerance of Angelonia Angustifolia with Various Ploidy Levels. HortScience.

[31] Tong S., Wang Y., Chen N., Wang D., Liu B., Wang W., Chen Y., Liu J., Ma T., Jiang Y. (2022). PtoNF-YC9-SRMT-PtoRD26 Module Regulates the High Saline Tolerance of a Triploid Poplar. Genome Biol..

[32] Chao D.-Y., Dilkes B., Luo H., Douglas A., Yakubova E., Lahner B., Salt D.E. (2013). Polyploids Exhibit Higher Potassium Uptake and Salinity Tolerance in *Arabidopsis*. Science.

[33] Li X., Yu E., Fan C., Zhang C., Fu T., Zhou Y. (2012). Developmental, Cytological and Transcriptional Analysis of Autotetraploid *Arabidopsis*. Planta.

[34] Fu H., Zhao J., Ren Z., Yang K., Wang C., Zhang X., Elesawi I.E., Zhang X., Xia J., Chen C. (2022). Interfered Chromosome Pairing at High Temperature Promotes Meiotic Instability in Autotetraploid *Arabidopsis*. Plant Physiol..

[35] Yu Z., Haberer G., Matthes M., Rattei T., Mayer K.F.X., Gierl A., Torres-Ruiz R.A. (2010). Impact of Natural Genetic Variation on the Transcriptome of Autotetraploid *Arabidopsis thaliana*. Proc. Natl. Acad. Sci. USA.

[36] Soltis D.E., Misra B.B., Shan S., Chen S., Soltis P.S. (2016). Polyploidy and the Proteome. Biochim. Biophys. Acta (BBA)-Proteins Proteom..

[37] Rodriguez M.C., Mehta D., Tan M., Uhrig R.G. (2021). Quantitative Proteome and PTMome Analysis of *Arabidopsis thaliana* Root Responses to Persistent Osmotic and Salinity Stress. Plant Cell Physiol..

[38] Ravi M., Chan S.W.L. (2010). Haploid Plants Produced by Centromere-Mediated Genome Elimination. Nature.

[39] Aguirre M., Loperfido D., Ezquer I. (2025). Decoding Hybridization Barriers: The Molecular and Genetic Orchestration of the Triploid Block in Arabidopsis Thaliana. BMC Plant Biol..

[40] Dilkes B.P., Spielman M., Weizbauer R., Watson B., Burkart-Waco D., Scott R.J., Comai L. (2008). The Maternally Expressed WRKY Transcription Factor TTG2 Controls Lethality in Interploidy Crosses of Arabidopsis. PLoS Biol..

[41] Ripoll J., Bertin N., Bidel L.P.R., Urban L. (2016). A User’s View of the Parameters Derived from the Induction Curves of Maximal Chlorophyll a Fluorescence: Perspectives for Analyzing Stress. Front. Plant Sci..

[42] Strasser R.J., Tsimilli-Michael M., Srivastava A. (2004). Analysis of the Chlorophyll a Fluorescence Transient. Advances in Photosynthesis and Respiration.

[43] Jedmowski C., Ashoub A., Momtaz O., Brüggemann W. (2015). Impact of Drought, Heat, and Their Combination on Chlorophyll Fluorescence and Yield of Wild Barley (*Hordeum spontaneum*). J. Bot..

[44] Carillo P., Gibon Y. (2011). Protocol: Extraction and Determination of Proline. PrometheusWiki.

[45] Faurobert M., Pelpoir E., Chaïb J., Thiellement H., Zivy M., Damerval C., Méchin V. (2007). Phenol Extraction of Proteins for Proteomic Studies of Recalcitrant Plant Tissues. Plant Proteomics: Methods and Protocols.

[46] Pavoković D., Poljuha D., Horvatić A., Ljubešić N., Hagège D., Krsnik-Rasol M. (2012). Morphological and Proteomic Analyses of Sugar Beet Cultures and Identifying Putative Markers for Cell Differentiation. Plant Cell Tiss. Organ. Cult..

[47] Pavoković D., Križnik B., Krsnik-Rasol M. (2012). Evaluation of Protein Extraction Methods for Proteomic Analysis of Non-Model Recalcitrant Plant Tissues. Croat. Chem. Acta.

[48] Suter L., Widmer A. (2013). Phenotypic Effects of Salt and Heat Stress over Three Generations in *Arabidopsis thaliana*. PLoS ONE.

[49] Sharkey T.D. (2005). Effects of Moderate Heat Stress on Photosynthesis: Importance of Thylakoid Reactions, Rubisco Deactivation, Reactive Oxygen Species, and Thermotolerance Provided by Isoprene. Plant Cell Environ..

[50] Shu S., Guo S., Sun J., Yuan L. (2012). Effects of Salt Stress on the Structure and Function of the Photosynthetic Apparatus in Cucumis Sativus and Its Protection by Exogenous Putrescine. Physiol. Plant..

[51] Del Pozo J.C., Ramirez-Parra E. (2014). Deciphering the Molecular Bases for Drought Tolerance in *Arabidopsis* Autotetraploids. Plant Cell Environ..

[52] Oyoshi K., Katano K., Yunose M., Suzuki N. (2020). Memory of 5-Min Heat Stress in *Arabidopsis thaliana*. Plant Signal. Behav..

[53] Kadir S., Von Weihe M., Al-Khatib K. (2007). Photochemical Efficiency and Recovery of Photosystem II in Grapes After Exposure to Sudden and Gradual Heat Stress. J. Amer. Soc. Hort. Sci..

[54] Fischer S., Flis P., Zhao F.-J., Salt D.E. (2022). Transcriptional Network Underpinning Ploidy-Related Elevated Leaf Potassium in Neo-Tetraploids. Plant Physiol..

[55] Verbruggen N., Hermans C. (2008). Proline Accumulation in Plants: A Review. Amino Acids.

[56] Verbruggen N., Villarroel R., Van Montagu M. (1993). Osmoregulation of a Pyrroline-5-Carboxylate Reductase Gene in *Arabidopsis thaliana*. Plant Physiol..

[57] Vives-Peris V., Gómez-Cadenas A., Pérez-Clemente R.M. (2017). Citrus Plants Exude Proline and Phytohormones under Abiotic Stress Conditions. Plant Cell Rep..

[58] Yaish M.W. (2015). Short Communication Proline Accumulation Is a General Response to Abiotic Stress in the Date Palm Tree (*Phoenix dactylifera* L.). Genet. Mol. Res..

[59] Yoshiba Y., Kiyosue T., Nakashima K., Yamaguchi-Shinozaki K., Shinozaki K. (1997). Regulation of Levels of Proline as an Osmolyte in Plants under Water Stress. Plant Cell Physiol..

[60] Wu Z., Liang J., Wang C., Zhao X., Zhong X., Cao X., Li G., He J., Yi M. (2018). Overexpression of Lily *HsfA3s* in Arabidopsis Confers Increased Thermotolerance and Salt Sensitivity via Alterations in Proline Catabolism. J. Exp. Bot..

[61] Hua X.J., Van De Cotte B., Van Montagu M., Verbruggen N. (2001). The 5′ Untranslated Region of the At-P5R Gene Is Involved in Both Transcriptional and Post-transcriptional Regulation. Plant J..

[62] Nanjo T., Kobayashi M., Yoshiba Y., Kakubari Y., Yamaguchi-Shinozaki K., Shinozaki K. (1999). Antisense Suppression of Proline Degradation Improves Tolerance to Freezing and Salinity in *Arabidopsis thaliana*. FEBS Lett..

[63] El Moukhtari A., Cabassa-Hourton C., Farissi M., Savouré A. (2020). How Does Proline Treatment Promote Salt Stress Tolerance During Crop Plant Development?. Front. Plant Sci..

[64] Hemmati H., Gupta D., Basu C., Pandey G.K. (2015). Molecular Physiology of Heat Stress Responses in Plants. Elucidation of Abiotic Stress Signaling in Plants.

[65] Rocco M., Arena S., Renzone G., Scippa G.S., Lomaglio T., Verrillo F., Scaloni A., Marra M. (2013). Proteomic Analysis of Temperature Stress-Responsive Proteins in *Arabidopsis thaliana* Rosette Leaves. Mol. BioSyst..

[66] Kosová K., Vítámvás P., Prášil I.T., Renaut J. (2011). Plant Proteome Changes under Abiotic Stress—Contribution of Proteomics Studies to Understanding Plant Stress Response. J. Proteom..

[67] Han F., Chen H., Li X.-J., Yang M.-F., Liu G.-S., Shen S.-H. (2009). A Comparative Proteomic Analysis of Rice Seedlings under Various High-Temperature Stresses. Biochim. Biophys. Acta (BBA)-Proteins Proteom..

[68] Law R.D., Crafts-Brandner S.J. (2001). High Temperature Stress Increases the Expression of Wheat Leaf Ribulose-1,5-Bisphosphate Carboxylase/Oxygenase Activase Protein. Arch. Biochem. Biophys..

[69] Zou J., Liu C., Chen X. (2011). Proteomics of Rice in Response to Heat Stress and Advances in Genetic Engineering for Heat Tolerance in Rice. Plant Cell Rep..

[70] Lim C.J., Yang K.A., Hong J.K., Choi J.S., Yun D.-J., Hong J.C., Chung W.S., Lee S.Y., Cho M.J., Lim C.O. (2006). Gene Expression Profiles during Heat Acclimation in *Arabidopsis thaliana* Suspension-Culture Cells. J. Plant Res..

[71] Zhang H.M., Zhang L.S., Liu L., Zhu W.N., Yang W.B. (2013). Changes of Dehydrin Profiles Induced by Drought in Winter Wheat at Different Developmental Stages. Biol. Plant..

[72] Port A., Clapco S., Duca M., Burcovschi I., Joiţa-Păcureanu M. (2023). Accumulation of Dehydrin Transcripts Correlates with Tolerance to Drought Stress in Sunflower. Rom. Agric. Res..

[73] Lin B.-L., Wang J.-S., Liu H.-C., Chen R.-W., Meyer Y., Barakat A., Delseny M. (2001). Genomic Analysis of the Hsp70 Superfamily in *Arabidopsis thaliana*. Cell Stress Chaperones.

[74] Su P.-H., Li H. (2008). Arabidopsis Stromal 70-kD Heat Shock Proteins Are Essential for Plant Development and Important for Thermotolerance of Germinating Seeds. Plant Physiol..

[75] Qazi H.A., Jan N., Ramazan S., John R. (2019). Protein Modification in Plants in Response to Abiotic Stress. Protein Modificomics.

[76] Barta C., Dunkle A.M., Wachter R.M., Salvucci M.E. (2010). Structural Changes Associated with the Acute Thermal Instability of Rubisco Activase. Arch. Biochem. Biophys..

[77] Qu Y., Sakoda K., Fukayama H., Kondo E., Suzuki Y., Makino A., Terashima I., Yamori W. (2021). Overexpression of Both Rubisco and Rubisco Activase Rescues Rice Photosynthesis and Biomass under Heat Stress. Plant Cell Environ..

[78] Salvucci M.E. (2007). Association of Rubisco Activase with Chaperonin-60: A Possible Mechanism for Protecting Photosynthesis during Heat Stress. J. Exp. Bot..

[79] Pang Q., Chen S., Dai S., Chen Y., Wang Y., Yan X. (2010). Comparative Proteomics of Salt Tolerance in *Arabidopsis thaliana* and *Thellungiella halophila*. J. Proteome Res..

[80] Igarashi D., Tsuchida H., Miyao M., Ohsumi C. (2006). Glutamate:Glyoxylate Aminotransferase Modulates Amino Acid Content during Photorespiration. Plant Physiol..

[81] Verslues P.E., Kim Y.-S., Zhu J.-K. (2007). Altered ABA, Proline and Hydrogen Peroxide in an Arabidopsis Glutamate:Glyoxylate Aminotransferase Mutant. Plant Mol. Biol..

